# The effectiveness of pharmacological agents for the treatment of uveitic macular oedema (UMO): a systematic review protocol

**DOI:** 10.1186/s13643-016-0203-y

**Published:** 2016-02-13

**Authors:** Mohammad O. Tallouzi, David J. Moore, Melanie Calvert, Philip I. Murray, Nicholas Bucknall, Alastair K. Denniston

**Affiliations:** Institute of Applied Health Research, College of Medical and Dental Sciences, University of Birmingham, Edgbaston, Birmingham, B15 2TT UK; Institute of Immunology and Immunotherapy, College of Medical and Dental Sciences, University of Birmingham, Birmingham, UK; Birdshot Uveitis Society representative, Birmingham, UK; Department of Ophthalmology, Queen Elizabeth hospital, University Hospitals Birmingham NHS Foundation Trust, Edgbaston, Birmingham, B15 2WB UK

**Keywords:** Systematic review, Macular oedema, Macular edema, Uveitis, Management, Pharmacological agents, Meta-analysis

## Abstract

**Background:**

Macular oedema (MO) describes the accumulation of fluid in the central part of the retina, known as the ‘macula’ which provides central vision. MO is the leading cause of sight loss in patients with intraocular inflammation (uveitis). There is a lack of consensus over the treatment of uveitic macular oedema (UMO). The proposed systematic review will evaluate the evidence on the effectiveness of pharmacological agents used to treat UMO. All systemic, local, or topical pharmacological agents will be included.

**Method/design:**

Standard systematic review methodology will be employed to identify, select and extract data from comparative studies (randomised/non-randomised trials and observational studies) of the pharmacological interventions in patients with UMO. Searches will be conducted through bibliographic databases (Cochrane Library, MEDLINE, EMBASE and CINAHL) and clinical trials registers. No restriction will be placed on either language or year of publication. Translation of non-English language articles will be undertaken to minimise selection bias. The primary outcome of interest will be best corrected visual acuity and secondary outcomes will be adverse events, health-related quality of life, assessment of UMO using central macular thickness (e.g. by optical coherence topography (OCT)), clinical and angiographic assessment of UMO, clinical estimation of vitreous haze. Risk of bias assessment appropriate to each study design will be undertaken. Data will be grouped by comparison, tabulated and narratively synthesised. Meta-analysis will be undertaken where clinical and methodological homogeneity exists. Subgroup and sensitivity analyses, also network analyses and intra/inter-pharmacological class analyses will be undertaken where deemed appropriate.

**Discussion:**

A number of published studies have investigated the effectiveness of the pharmacological agents used to treat UMO. However, there is no recent systematic review that synthesises this evidence. This systematic review will analyse the effectiveness of systemic, local and topical therapies to treat UMO. The findings will provide important evidence to inform clinical and health policy decision-making for the treatment of UMO.

**Systematic review registration:**

Prospero CRD42015019170

**Electronic supplementary material:**

The online version of this article (doi:10.1186/s13643-016-0203-y) contains supplementary material, which is available to authorized users.

## Background

Uveitis describes a group of disorders characterised by intraocular inflammation. Uveitis is the fifth commonest cause of visual loss in the developed world and accounts for about 10–15 % of total blindness [1, 2] and up to 25 % in the developing world [3, 4]. Although uveitis may affect any age group, it peaks in the working age population with no significant gender difference [5]. The annual incidence of uveitis is estimated at 14–50 per 100,000 with a prevalence of around 38–200 per 100,000 general population [1, 2, 5, 6].

Uveitis has a disproportionately high impact in terms of years of potential vision loss and economic effects because it often strikes at a younger age than common age-related eye disorders such as cataract, age-related macular degeneration and glaucoma [1].

Uveitis may be classified anatomically as anterior uveitis, intermediate uveitis, posterior uveitis or panuveitis [7, 8]. The leading cause of sight loss in patients with uveitis is macular oedema and known in this context as uveitic macular oedema (UMO) [1, 9]. Macular oedema (MO) describes the accumulation of fluid in the retina (the light-sensitive inner-lining of the eye) in the area that provides central vision known as the ‘macula’ [10]. MO is more common in those forms of uveitis which affect the more posterior structures in the eye, namely intermediate, posterior or panuveitis; collectively, these are sometimes referred to as posterior segment-involving uveitis. MO can also occur in association with anterior uveitis [11].

Macular oedema accounts for 41 % of visual impairment and 29 % of blindness in uveitis [6, 12]. In the Multicentre Uveitis Steroid Treatment (MUST) trial of systemic corticosteroid vs a fluocinolone acetonide implant in non-infectious intermediate, posterior and panuveitis, it was noted that low vision (best corrected visual acuity (BCVA) worse than 20/40) was present in 50 % of recruited patients and legal blindness (BCVA of 20/200 or worse) in 16 %, with cystoid macular oedema being present in 38 % of eyes with similar distribution across intermediate uveitis, posterior uveitis and panuveitis [13].

The impact of UMO on visual acuity is usually assessed using standard distance visual acuity charts, either using a Snellen chart or Early Treatment Diabetic Retinopathy Study (ETDRS) chart. Acuities from Snellen charts are usually reported in metres in the UK and feet in the USA. Acuities from ETDRS charts are usually reported either as ‘number of letters read’ or converted into a LogMAR fraction. Although certain visual acuities are considered to be equivalent (e.g. 0.0 LogMAR = 6/6 UK Snellen = 20/20 US Snellen), due to intrinsic differences between the charts, it is recognised that these equivalences are approximate [11]. Although the Snellen chart is still widely used in clinical practice, most trials use ETDRS charts due to various methodological advantages. Traditionally, MO has been assessed clinically using stereoscopic slit-lamp fundus bio-microscopy and fluorescein angiography, an invasive procedure requiring intravenous dye and stereo photography imaging testing [14]. More recently, a non-invasive imaging technique, optical coherence tomography (OCT), has become a standard clinical practice in the follow-up of UMO and monitoring treatment response [15, 16]. OCT may be more sensitive than clinical measures in detecting the presence of UMO and provides accurate measures of the structural changes in terms of macular thickness [17].

The treatment of UMO is a major priority in tackling sight loss in uveitis and will be the focus of this study. Corticosteroids are the mainstay of treatment for UMO [10], with alternative routes of administration: systemic (oral, intravenous and intramuscular); local which includes peri-ocular injection (sub-Tenon and orbital floor injection) and intraocular (intra-vitreal injection or implant) [18, 19]. ‘Second line’ therapies are typically immunomodulatory and include T cell inhibitors (e.g. ciclosporine, tacrolimus), antimetabolites (e.g. azathioprine, methotrexate, mycophenolate Mofetil), alkylating agents (e.g. cyclophosphamide) and biological agents (e.g. interferons, antitumor necrosis factor (anti-tumour necrosis factor (TNF)) agents) [20–23]. Most of these agents are only used systemically (oral, intravenous or subcutaneous), while intra-vitreal use has been reported for both methotrexate and anti-TNF agents [22–25]. Other treatments that have been used in UMO include the oral carbonic anhydrase inhibitor (acetazolamide), and intra-vitreal anti-vascular endothelial growth factor (anti-VEGF) agents [10, 26].

Whilst there have been narrative reviews on the management of UMO, [10] a scoping search of Cochrane library, MEDLINE, identified that only one systematic review has been undertaken [16]. That review aimed to cover all pharmacological interventions for UMO and had fairly comprehensive searches, not restricted by language or year of publication, undertaken up to late 2011. The review only included RCTs of which nine were reviewed. Limitations to this work include a lack of steps to minimise bias in the review process, and there are potential concerns over transparency in reporting as the review does not meet PRISMA (Preferred Reporting Items for Systematic Reviews and Meta-analyses standards). The review highlights the availability of trials across the classes of pharmacological interventions yet meta-analysis was limited by heterogeneity and availability of data. The authors also noted that relevant work was ongoing, with more than 10 clinical trials related to UMO in progress at the time of that review.

Further, scoping work has suggested a role for non-RCT evidence in this field. Scoping searches suggest that RCTS in this field are likely to be small in size and may have shorter periods of follow-up than non-RCTs. Non-RCTs may therefore be more suitable for the detection of adverse events. There is thus value in including the non-RCT body of evidence in any new systematic review.

Although there is a wide range of treatment options available for UMO, there are currently no consensus guidelines to direct treatment in this field. This may lead to uncertainty for patients, clinicians and healthcare providers. It is timely to review the literature in order to evaluate and summarise the available evidence for the pharmacological agents used for the treatment of UMO, which may form the basis of evidence-based clinical recommendations. Identifying the most effective treatment for ocular inflammatory disease is the number one priority for research in inflammatory eye disease [27].

## Methods/design

### Aim

The aim is to assess the effectiveness of the available pharmacological therapies used in the treatment of UMO. The aim will be achieved by conducting a systematic review of studies:

- Comparing a pharmacological agent to the non-use of a pharmacological agent.
- Comparing a pharmacological agent to the same or another pharmacological agent.

Standard protocol-driven systematic review methods will be used.

The systematic review protocol has been registered with the international prospective register of systematic reviews (PROSPERO) database ref (CRD42015019170) and has been reported according to the PRISMA-P guidlines (see Additional file 1).

### Searches

The following sources will be searched.

- Bibliographic databases of published studies
- MEDLINE, MEDLINE in process (Ovid).
- EMBASE (Ovid).
- CINAHL (EBSCO)
- The Cochrane Library (CENTRAL Register of Controlled Trials, Cochrane Database of Systematic Reviews, Database of Abstracts of Reviews of Effects and Health Technology Assessment database).

The search strategy will combine index and free text terms for the condition (MO) and the disease context (uveitis) where possible. A sample research strategy from MEDLINE is provided in Table 1, and this strategy will be adapted for the use in each bibliographic database.

**Table 1:** MEDLINE sample search strategy for uveitic macular edema

Count	Searches
1	Exp Macular Edema/
2	(macular adj2 edema). ti,ab.
3	(macular adj2 edema). ti,ab.
4	1 or 2 or 3
5	Exp Uveitis/
6	Uveit$.ti,ab.
7	5 or 6
8	4 and 7

Identified systematic reviews will be used to check if all relevant primary studies are identified.

- Registers of Clinical trials
- Clinicaltrials.gov. www.clinicaltrials.gov.
- International Standard Randomised Controlled Trials Number (ISRCTN database). www.contolled-trials.com
- WHO International Clinical Trials Registry Platform (ICTRP portal). www.who.int/ictrp/en/.
- UK Clinical Research Network (UKCRN). www.ukcrc.org
- Abstract and conference proceedings
- British Library’s ZETOC.
- Conference proceedings Citation Index (Web of Science).
- Dissertations, theses
- British library Ethos
- ProQuest. www.proquest.com
- Grey literature
- OpenGrey. www.opengrey.eu

These sources will be searched in a more iterative way as complex search strategies may not be able to be used; therefore, keywords/phrases based on macular edema/oedema and uveitis will be employed.

There will be no restriction placed on either language or year of publication; however, for conference abstracts, only those within 3 years of the search date will be considered. The literature search results will be entered onto EndNote ×7 (Thomson Reuters) to facilitate the removal of duplicate records, study selection, recording decisions and references.

### Selection criteria

The following criteria will be used to select studies for review:

- Study design
- Randomised controlled trials (RCTs) and other comparative studies where the comparator group is from a concurrent time period (e.g. non-randomised controlled trials, comparative observational studies).
- Participants
- Participants of any age, gender or ethnicity with a diagnosis of UMO.

Studies on a population broader than UMO will only be included if data specific for the UMO subgroup is reported separately.

- Intervention and comparator
- Comparing any pharmacological agent to no use of a pharmacological agent.
- Comparing any pharmacological agent to the same or another pharmacological agent.
- Outcomes
- Outcomes will not be used for study selection. However, clinical- and patient-reported outcomes are considered important for the aims of the review.
- Primary outcome.
- Best corrected visual acuity.
- Secondary outcome
- Adverse events.
- Health-related quality of life.
- Central macular thickness (e.g. by OCT)
- Angiographic assessment of UMO
- Clinical assessment of UMO
- Clinical estimation of vitreous haze
- Clinical estimation of anterior chamber cells

### Selection process

The study selection process will be conducted in two stages:

- First, title and abstract of the identified articles will be screened in order to remove irrelevant records. Articles that obviously do not meet the selection criteria will be excluded.
- Second, the full text of the potential relevant articles will be retrieved and assessed against all the selection criteria.

At both stages, two reviewers will independently assess articles with any disagreements resolved by discussion and, if required, referral to a third reviewer. Both stages of the selection process will be piloted and if necessary modified. The study selection processes will be illustrated using a PRISMA flow diagram and details of articles excluded at the full text stage will be recorded along with the reason for exclusion [28].

Translation in part or wholly of non-English language articles will be undertaken to aid study selection and analysis.

### Data extraction

Two authors will independently extract data from the included publications. Any discrepancies will be resolved through discussion and referral to a third reviewer if needed. A standardised piloted data extraction form will be used. Study authors may be contacted if further information is required. For each study, the following information (but not limited to) will be extracted.

- Study characteristics
- Authors—publication year—title and journal
- Study design
- Setting
- Sample size
- Length of follow-up
- Analysis
- Participant characteristics
- Patient selection/recruitment criteria
- Patients’ characteristics (demographic data, number, age, gender, socioeconomic status and ethnicity).
- Type of uveitis (anatomical categorisation, syndrome/aetiological classification)
- Comorbidity
- Co-medication
- Intervention and comparator
- Pharmacological agents
- Regimen (dose, frequency of administration, route of administration)
- Comparator details
- Any difference in underlying care between treatment groups
- Outcomes and findings
- Outcomes measured and results for each outcome including precision and statistical test results.
- Completeness of follow-up for each outcome

### Quality assessment

Quality assessment of all included articles will be undertaken by two reviewers independently with disagreements resolved by discussion and referral to a third reviewer if required.

The risk of bias tool from the Cochrane Handbook will be used for RCTs [29]. For non-randomised controlled trials, the domains in the risk of bias tool for RCTs will be used (accepting that criteria for randomisation and possibly concealment of allocation are not relevant). For prospective controlled observational studies, the guidelines outlined in Chapter 13 of the Cochrane Handbook will be followed [29]. The domains in the risk of bias tool for RCTs can be used as a minimum assessment (again accepting that the studies are not randomised). The most relevant criteria for assessment in this area are likely to relate to how the groups were selected, differences in patient characteristics, loss to follow-up and biases and confounding in outcome assessment. Any case controlled studies/analyses will be assessed based on the Newcastle-Ottawa scale [30].

### Analysis

Studies will be grouped by each intervention and comparison, with data tabulated and a narrative synthesis of evidence conducted for each outcome of relevance to the review.

Assessment of clinical and methodological heterogeneity will be employed to determine whether for each comparison for each outcome, studies are sufficiently similar to ensure data pooling by meta-analysis is appropriate and whether a random effect or fixed effect model is the most appropriate [31]. The *I*^2^ statistic (which gives the percentage of the total variability in the data due to between-study heterogeneity) and the tau-squared statistic (which gives an estimate of the between-study variance) will be reported where appropriate. Data from differing study designs will not be pooled together. For each meta-analysis containing 10 or more studies, the likelihood publication bias will be investigated and funnel plot will be constructed [32].

It is expected that multiple time point data will be available within the same study and between studies. Nominally, data will be categorised in each analysis into the following groups based on follow-up period: ≤3 months, >3 and ≤6 months and >6 months post interventions. Within the last category, further division may be considered to assess longer-term data.

Results for some outcomes are likely to be presented using a number of different measure/statistics within the same study and/or between studies. For example, visual acuity may be reported in metres or feet (from Snellen charts), a LogMAR score, number of letters or lines read (from ETDRS charts), and the change in acuity may be reported as a change in any one of these indices or categorised against a threshold, e.g. proportion of subjects with change greater or equal to a specific number of lines/letters read [11]. Visual acuity can therefore be considered as continuous data (e.g. group mean LogMAR score), some as discrete data (e.g. number of lines read) and some as dichotomous data (e.g. proportion of patients reading x lines, or proportion with a LogMAR score greater than *y*). The first and last are likely to be the most common data. Conversion of data between formats to maximise the data available for each analyses will be considered (for example if the type of chart is known, letters might be able to be converted to lines; LogMAR score and letters interchanged; Snellen UK, US and ETDRS data approximated. Any conversion of data will be undertaken with due caution and with regard known issues [11]. The impact of any converted data on findings will be explicitly acknowledged.

Continuous data (e.g. health related quality of life) from the same scale will be pooled using mean difference and from a different scale where tools are considered to be assessing the same underlying features, standardised mean difference will be used. Further, subgroup analysis will be considered where deemed appropriate. Where data allows, such analysis could include grouping by clinical and anatomical classification to the type of uveitis (anterior, intermediate, posterior and pan) and route of administration of the intervention.

Direct comparison of interventions will be undertaken via included head-to-head studies where these are available. For included randomised controlled trials, the potential for network meta-analysis or adjusted indirect comparison will be explored. It may be possible to estimate the relative effect of the different pharmacological agents if sufficient studies exist to inform the network. The ability to undertake network meta-analyses/adjusted indirect treatment comparisons will be dependent on a number of key assumptions (e.g. the homogeneity, similarity and consistency assumptions) [33, 34].

Based on all the above, as the interventions fall into five classes of agent (corticosteroids, T cell inhibitors, antimetabolites, alkylating agents and biological agents) evidence of effect of agents within each class will be discussed to report on the consistency and magnitude of the class effect. Finally, any inference of comparisons between classes will be considered.

### Reporting

The review and its findings will be reported in accordance to the PRISMA guidelines [28]. The strengths and weaknesses of the review methods and the available evidence will be discussed in relation to the internal and external validity of the findings. The implications of the review findings will be discussed in the context of current and future clinical practice related to UMO and the future research agenda.

### Discussion and potential impact

UMO is a major cause of blindness in the working age population. However, there is a wide variation in treatment reflecting limitations in primary data and a lack of national guidelines or consensus statements. This review will systematically and comprehensively retrieve evidence from a wide range of sources to evaluate the pharmacological treatment of the UMO. Furthermore, this review will provide valuable information regarding the effectiveness of pharmacological agents.

We hope this review will provide a clear reference point for ophthalmologist and decision makers. The revealed evidence will aid standardisation of clinical practice on the most effective therapies to improve outcomes for patients and help minimise harm from inappropriate therapies. Furthermore, this review is timely due to the recent availability of novel local therapies which have been approved by UK National Institute for Health and Care Excellence (NICE) for other forms of MO, but whose role in UMO is not yet established.

## Additional file

Additional file 1.
**PRISMA-P 2015 checklist: recommended items to include in a systematic review protocol.** ^**a**^ (PDF 153 kb)

## Abbreviations

Anti-VEGF: anti-vascular endothelia growth factor
BCVA: best corrected visual acuity
CENTRAL: Cochrane Central Register of Controlled Trials
CINAHL: Cumulative Index to Nursing and Allied Health Literature
ETDRS: Early Treatment Diabetic Retinopathy Study
MEDLINE: Medical Literature Analysis and Retrieval System Online
NICE: National Institute for Health and Care Excellence
OCT: optical coherence topography
PRISMA: Preferred Reporting Items for Systematic Reviews and Meta-analyses
TNF: tumour necrosis factor
UMO: uveitic macular oedema
